# A Novel On-Chip Impedance Sensor for the Detection of Particle Contamination in Hydraulic Oil

**DOI:** 10.3390/mi8080249

**Published:** 2017-08-14

**Authors:** Hongpeng Zhang, Lin Zeng, Huaibo Teng, Xingming Zhang

**Affiliations:** 1 Marine Engineering College, Dalian Maritime University, Dalian 116026, China; bobzl@dlmu.edu.cn (L.Z.); tenghuaibo1989@dlmu.edu.cn (H.T.); 2 School of Naval Architecture and Ocean Engineering, Harbin Institute of Technology, Weihai 264209, China; zhxm@hit.edu.cn

**Keywords:** particle contamination, hydraulic oil, inductive sensor, capacitive sensor, microfluidic chip

## Abstract

A novel impedance sensor based on a microfluidic chip is presented. The sensor consists of two single-layer coils and a straight micro-channel, and can detect, not only ferromagnetic and non-ferromagnetic particles in oil as an inductive sensor, but also, water droplets and air bubbles in oil as a capacitive sensor. The experiments are carried out at different excitation frequencies, number of coil turns and particle sizes. For the inductance detection, the inductance signals are found to increase with the excitation frequency and the noise is constant; both the inductance signals and the noise increase with the number of coil turns, but because the noise increases at a faster rate than the signal, the signal-to-noise ratio decreases with the number of coil turns. We demonstrate the successful detection of 40 μm iron particles and 110 μm copper particles using the coil with 20 turns at the excitation frequency of 2 MHz. For the capacitance detection, capacitance signals decrease with the excitation frequency and the noise is constant; the capacitance signals decrease with the number of coil turns, while the noise increases, thus, the signal-to-noise ratio decreases with the number of coil turns. We can detect 100 μm water droplets and 180 μm bubbles successfully using the coil with 20 turns at the excitation frequency of 0.3 MHz.

## 1. Introduction

The hydraulic system has become an important part of large mechanical equipment. With the development of hydraulic technology towards high speed, high pressure and small size, many kinds of control elements with high sensitivity and complicated structure are introduced into hydraulic systems. As a consequence, there are more stringent requirements for the cleanliness of hydraulic oil. Statistical data show that more than 75% of the failures in hydraulic systems are caused by particle pollutants in hydraulic oil [1]. It is therefore necessary to carry out the detection of contaminants in oil to prevent failures in mechanical equipment.

A lot of information related to the working states of mechanical equipment appears in hydraulic oil when the hydraulic system is running, such as metal wear debris, water droplets and air bubbles in oil. During normal conditions, metal wear debris in hydraulic oil have a constant concentration and smaller size, usually in the range of 10–20 μm. However, the size of particles will increase to 50–100 μm and the concentration will increase sharply as well when abnormal wear happens [2,3]. Water moisture in oil will greatly accelerate the oxidation and corrosion process of mechanical equipment and result in aggregation of particle contamination [4]. The air bubbles in oil can cause cavitation erosion phenomenon which will reduce the service life of the hydraulic element due to the resulting temperature rise and oil deterioration in the system [5,6].

At present the laboratory analysis is still the main method for the detection of hydraulic oil. This type of methods can make a comprehensive analysis in the physical and chemical states of oil. But it relies on a variety of laboratory equipment and complexity in operation [7]. Moreover, it needs a long time and cannot achieve online detections.

In recent years, a series of methods for the rapid detection of contaminants in oil have been developed [8], including optical detection, acoustic detection, inductance detection and capacitance detection. Optical detection [9,10] has high detection accuracy but cannot distinguish between the properties of wear debris and is affected by the light transmittance of oil. Acoustic detection [3,11] takes advantage of a reflected sound wave to detect particles in oil; it is affected by the environment noise and the temperature change of the oil. Inductance detection [12,13,14,15,16] can make a distinction between ferromagnetic and non-ferromagnetic particles in oil and has high stability in the detection process. However, this method is not sensitive to water droplets and air bubbles in oil, and the detection accuracy is lower than that of the methods above. According to the different relative permittivity of the medium between two plates, capacitance detection [17,18,19] can detect water droplets and air bubbles in oil. However, this method cannot distinguish metal wear debris in oil. All these methods can only detect one or two kinds of contaminants in oil and are unable to achieve comprehensive detection. Recently, Zhu et al. has presented a method using an integrated device for oil detection [20]; it has three independent sensors responsible for detecting wear particles, water droplets and the viscosity of oil simultaneously. However, the structure and fabrication of this device are complicated due to the presence of three sensors.

Our group has recently demonstrated a double-coil inductive sensor based on a microfluidic chip [21]. Based on that work, we have developed in this paper a novel on-chip impedance sensor that can detect both the inductance signal and the capacitance signal. This sensor is composed of two single-layer coils that can not only distinguish between ferromagnetic and non-ferromagnetic particles in oil as an inductive sensor but also detect water droplets and air bubbles in oil as a capacitive sensor.

## 2. Chip Design and Fabrication

**Figure 1:**
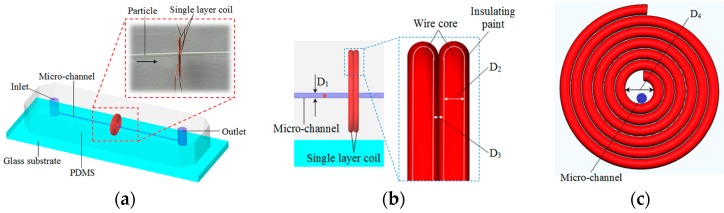
The design of the on-chip impedance sensor: (**a**) The overall design of the chip; (**b**) cross-section of the chip: D_1_ is the diameter of the micro-channel, D_2_ is the diameter of the coil wire core, D_3_ is the distance between the two single-layer coils; (**c**) a sketch of a single-layer coil: D_4_ is the inner diameter of the single-layer coil.

The impedance detection chip is shown in Figure 1. The chip mainly consists of an impedance sensor and a straight micro-channel. The impedance sensor comprises two single-layer coils, which cling together. The micro-channel, which is close to the inner wall, goes across the inner hole of the two coils. The diameter of the coil wire core D_2_ is 70 μm. There is a coat of insulating paint with a thickness of 10 μm around the coil wire core (see Figure 1b); thus, the distance between two coil wire cores D_3_ is 20 μm. The diameter of the coil inner hole D_4_ is 900 μm and the diameter of micro-channel D_1_ is 300 μm.

To fabricate the detection chip, a model of two coils and a straight micro-channel was made first. The two single-layer coils were each made of enamel copper wire and winded by a winding machine (Shili SRDZ23-1B, Zhongshan ShiLi Wire Winder Equipment Co., Ltd., Zhongshan, China); the winding direction of the two coils was the same and the number of turns is varied according to different experiments. They were then clung together. The straight micro-channel model was made of a copper rod with a diameter of 300 μm and a length of 7 cm. It was put into the coil inner hole close to the inner wall (see Figure 1c). After that, both the micro-channel model and the coils were fixed to a glass substrate using glue. The coils, micro-channel model and glass substrate formed a chip mold, upon which the liquid polydimethylsiloxane was poured. The chip mold was then placed in a thermostat with a temperature of 80 °C for 1 h. Finally, after the liquid polydimethylsiloxane was solidified, the straight micro-channel model was removed using pliers and the impedance detection chip fabrication was completed. The diameter of the micro-channel model equals to the diameter of the straight micro-channel.

## 3. Detection Mode

The impedance detection chip presented in this paper is both an inductive sensor and a capacitive sensor, it has two detection modes: inductance detection mode and capacitance detection mode. Details of the two modes are given blow.

### 3.1. Inductance Detection Mode

The single-layer coils will generate a magnetic field when they are energized by a high-frequency alternating current. When a metal particle passes through the detection area, on the one hand, the metal particle will be magnetized and the magnetic flux density will increase; on the other hand, the metal particle will generate an eddy current according to Lenz’s law, which will offset part of the original magnetic field and hence reduce the magnetic flux density. Due to the action of these two aspects, the magnetic field surrounding the metal particle will change, which can be captured by the coil, causing a change of the coil inductance [22,23].

**Figure 2:**
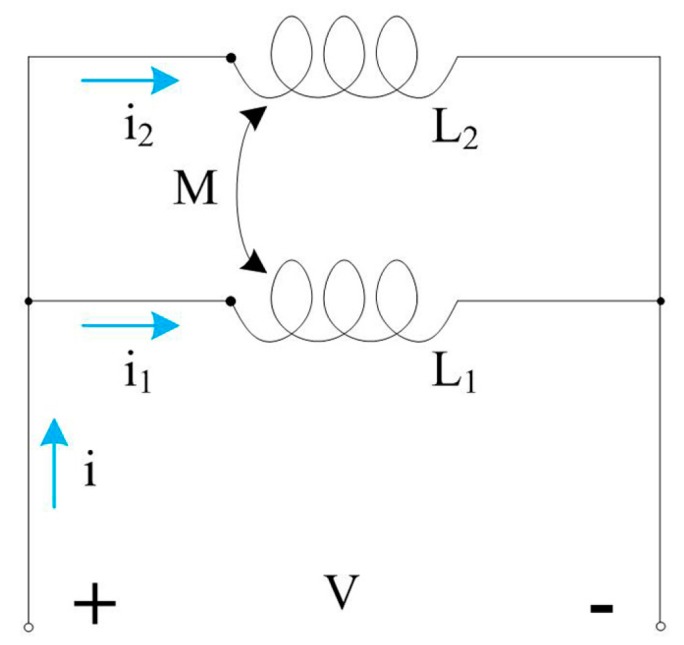
Schematic diagram of inductance detection: *L*_1_ and *L*_2_ are single-layer coil 1 and single-layer coil 2, respectively. *i*_1_ and *i*_2_ are the alternating currents that flow through the two coils.

The schematic diagram of the inductance detection is shown in Figure 2. It is a double-coil design with the two coils in parallel connection. The currents in the two coils, marked as coil 1 and coil 2 in Figure 2, flow in the same direction. The calculation of the inductance in a double coil is different from that in a single coil according to our prior research [21]. Because *L*_1_ = *L*_2_ = *L*, the equivalent inductance of a double coil can be obtained:

(1) $$L_{\mathrm{eq}}=\frac{L^{2}-M^{2}}{2L-2M}=\frac{L+M}{2}$$

From Equation (1), we know that the basic inductance of the double coil is lower than that of a single coil and hence has a higher sensitivity. According to our previous research results [24], the inductance change of a single solenoid coil caused by a metal particle at the center of the coil is given by

(2) ΔL=Im(ΔZmaxω)

And $\Delta Z_{\max}$ is given by

(3) $$\Delta Z_{\max}=j\pi \mu_{0}\omega n_{c}^{2}W^{2}K_{p}{\mathrm{lg}}^{2}(\frac{d+\sqrt{d^{2}+W^{2}}}{D+\sqrt{D^{2}+W^{2}}})$$

where *j*^2^ = −1, $\mu_{0}$ is the permeability of the vacuum, ω is the angular frequency of the excitation alternating current, *n*_c_ is the density of turns, that is

(4) $$n_{c}=\frac{2N}{W\left(D-d\right)}$$

And *N* is the number of turns of the single coil, *W* is the axial width of the coil (here *W = D*_2_ (the diameter of the coil wire core)), *d* is the diameter of the coil inner hole (here *d = D*_4_), *D* is the outer diameter of coil (that is *D* = *d* + 2*ND*_2_), and *K_p_* is the complex magnetization coefficient. As the difference in the relative permeability between ferromagnetic and non-ferromagnetic particles, *K*_p_ is different for the two types of particle.

For ferromagnetic metal particles, *K*_p_ is given by

(5) $$K_{P}=\frac{r^{3}}{2}\frac{\left(-r^{2}k^{2}+2\mu_{r}+1\right)\sin\left(2\mu_{r}+1\right)-rk\left(2\mu_{r}+1\right)\cos\left(rk\right)}{\left(r^{2}k^{2}+\mu_{r}-1\right)\sin\left(rk\right)-rk\left(\mu_{r}-1\right)\cos\left(rk\right)}$$

where *r* is the radius of particles and $\mu_{r}$ is the relative permeability of particles. For non-ferromagnetic metal particles (such as copper particles), $\mu_{r}\approx 1$.

Here, *k* is given by

(6) $$k=\sqrt{-j\omega \mu_{r}\mu_{0}\sigma }$$

where σ is the conductivity of particles. Thus, according to (1), the equivalent inductance change of a double coil can be obtained:

(7) $$\Delta L_{\mathrm{eq}}=\frac{\Delta L+\Delta M}{2}=\frac{\Delta L+\frac{M}{L}\Delta L}{2}$$

From Equations (3) to (7), we know that the inductance change of the double-coil presented in this paper is decided by the number of turns of the single coil *N*, the diameter of the coil wire core *W*, the diameter of the coil inner hole *d*, the radius of particles *r*, the relative permeability of particles $\mu_{r}$, the angular frequency of the excitation alternating current ω, the conductivity of particles σ and the mutual inductance of two coils *M*. The mutual inductance *M* is decided by the structure and the materials of the coil and the relative permeability of particles.

However, (7) is the inductance change caused by a metal particle which located at the center of the coil, but in this paper, the particle is located at the edge of the inner hole, and the references [25] and [26] show that radial position of metal particles has a significant effect on the coil inductance output, the inductance change caused by the particles at the edge of coil inner hole is larger than at the center of the coil inner hole. A simulation result [26] conducted by COMSOL software show that the inductance change caused by a 100 μm iron particle at the edge of the inner hole (300 μm from the center, which is the same distance as the microchannel in this paper) is 8.86 times larger than the inductance change at the center of the hole, and for a 150 μm iron particle, the increase in inductance change is about 7.91 times. So the equivalent inductance change of a double coil in this paper can be obtained:

(8) $$\Delta L_{\mathrm{eq}}=A\frac{\Delta L+\frac{M}{L}\Delta L}{2}$$

where *A* is the magnification of the inductance change caused by the particles away from the center of inner hole. The inductance change is only related to the properties and size of particles when the structural parameters of the double coil and the excitation alternating current are determinate. The relative permeability of ferromagnetic particles (such as iron particles) is much larger than 1. When a ferromagnetic particle passes through the detection area, its magnetization plays a major role; thus the magnetic induction intensity of the coil and the coil inductance will increase. In contrast, when a non-ferromagnetic particle (such as a copper particle) passes through the detection area, there is no magnetization and thus the eddy current plays a major role. Consequently, the magnetic induction intensity of the coil and the coil inductance will decrease. Therefore, the inductance detection mode can distinguish between ferromagnetic metal particles and non-ferromagnetic metal particles. Meanwhile, regarding water droplets and air bubbles, whose relative permeability is close to 1 and for which there is no eddy current, they will not generate any signal when passing the detection area.

### 3.2. Capacitance Detection Mode

**Figure 3:**
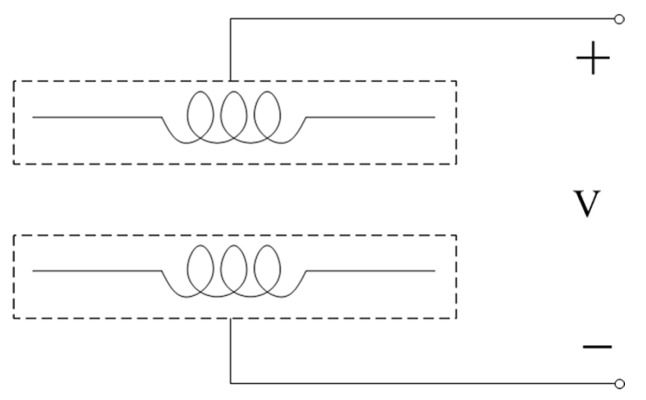
Schematic diagram of capacitance detection.

The schematic diagram of capacitance detection is shown in Figure 3. Here, the two single-layer coils are equivalent to a pair of capacitance plates; therefore, the double coil here is also a parallel plate ring capacitor. The capacitor can produce capacitance when the two plates are powered by an alternating current. The principle of capacitance detection is that when a particle passes through the capacitor, the permittivity of media between the plates will change; thus, the capacitance will be varied.

**Figure 4:**
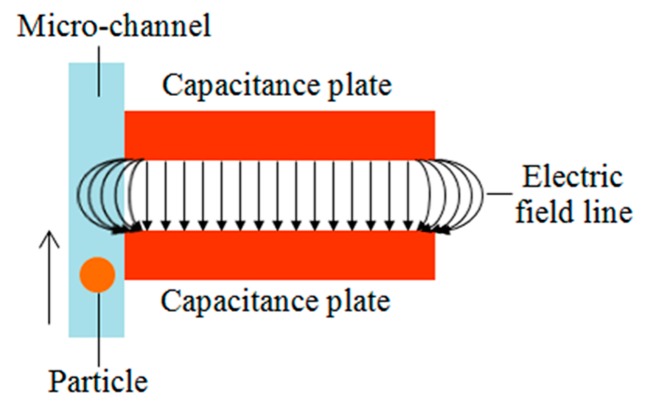
Electric field distribution of parallel plate capacitor.

Generally, in capacitance detection, the particle should pass through the gap between two plates. In our design the straight micro-channel is placed close to the inner wall of the double-coil (see Figure 1c); hence, the particles will pass through the inner hole of the coil (see Figure 1a) rather than the gap between two coils. Here, we use the fringe effect of the capacitor, as shown in Figure 4. In ideal conditions, the electric charge density on the plates is considered uniform and the fringing field of the edges is neglected. However, in actual conditions, the electric fields also distribute along the edges of plates, as shown in Figure 4. The electric fields in the edges become more complex compared with the center area.

When the fringe effect is taken into consideration, the electric charge densities also exist along the edges of the plates and the distribution is not uniform; thus, the calculation of the plate capacitance becomes complex. According to the existing literature, a few empirical formulas have been proposed using different methods to calculate the capacitance of the parallel plate capacitor when the fringe effect is under consideration [27,28,29]; however, there is no general method for calculating it accurately. Nishiyama et al. [30] presented an empirical formula to calculate the capacitance of parallel plate ring capacitors:

(9) $$C=\frac{Q}{V}=\frac{\pi \varepsilon t}{Vm^{2}}\sum_{i=1}^{m}\left[\left(2i-1\right)t+2mR\right]\;q_{i}$$

This formula is based on the boundary element method, the plate is divided into *m* ring boundary elements with equal width, in this paper, *m* equals to the number of turns, ε is the permittivity of media in the gap between plates, *V* is the potential difference between the two plates, *R* is the inner radius of the ring plate, *t* is the radial thickness of the ring (here, *t = ND*_2_), and *q* is the surface density charge of each element (detailed calculation process is shown in [30]). Therefore, the capacitance is only related to the permittivity of media when the structural parameters of the double coil and the excitation alternating current are determinate.

The relative permittivity of hydraulic oil is $\varepsilon_{o}$ = 2.6, when a particle passes through the detection area, the permittivity of media between the plates will change to $\varepsilon_{\mathrm{mix}}$. In this paper, the capacitor is excited by alternating current, the permittivity of media is not only related to the particle property, but also related to the excitation frequency, and so the complex permittivity of a material is used, which is given by

(10) $$\tilde{\varepsilon }=\varepsilon -j\frac{\sigma }{\omega }$$

where ε is the permittivity, σ is the conductivity, *j^2^* = −1 and ω is the angular frequency. The complex permittivity of hydraulic oil ${\tilde{\varepsilon }}_{o}$ is

(11) $${\tilde{\varepsilon }}_{o}=\varepsilon_{o}-j\frac{\sigma_{o}}{\omega }$$

The complex permittivity of particle ${\tilde{\varepsilon }}_{p}$ is

(12) $${\tilde{\varepsilon }}_{p}=\varepsilon_{p}-j\frac{\sigma_{p}}{\omega }$$

Based on Maxwell’s mixture equation [31], the equivalent complex permittivity of the mixture containing the particle and the oil is given by

(13) $${\tilde{\varepsilon }}_{\mathrm{mix}}={\tilde{\varepsilon }}_{o}\frac{1+2\Phi {\tilde{f}}_{\mathrm{CM}}}{1-\Phi {\tilde{f}}_{\mathrm{CM}}}$$

where ${\tilde{\varepsilon }}_{\mathrm{mix}}$ is the complex permittivity of the mixture, Φ is the volume fraction (ratio of the particle volume $V_{p}$ to the detection volume $V_{d}$), since the particle size is larger than the distance between two plates (90 μm, ignore the plate thickness), in the electric field area, Φ is given by

(14) $$\Phi =\frac{V_{p}}{V_{d}}=\frac{r^{2}}{{\left(R+t\right)}^{2}-R^{2}}$$

And ${\tilde{f}}_{\mathrm{CM}}$ is the Clausius–Mossotti factor, given by

(15) $${\tilde{f}}_{\mathrm{CM}}=\frac{{\tilde{\varepsilon }}_{p}-{\tilde{\varepsilon }}_{o}}{{\tilde{\varepsilon }}_{p}+2{\tilde{\varepsilon }}_{o}}$$

From (14) and (15), ${\tilde{\varepsilon }}_{\mathrm{mix}}$ can be obtained:

(16) $${\tilde{\varepsilon }}_{\mathrm{mix}}={\tilde{\varepsilon }}_{o}\frac{3V_{d}\left({\tilde{\varepsilon }}_{p}+2{\tilde{\varepsilon }}_{o}\right)+8\pi r^{3}\left({\tilde{\varepsilon }}_{p}-{\tilde{\varepsilon }}_{o}\right)}{3V_{d}\left({\tilde{\varepsilon }}_{p}+2{\tilde{\varepsilon }}_{o}\right)-4\pi r^{3}\left({\tilde{\varepsilon }}_{p}-{\tilde{\varepsilon }}_{o}\right)}$$

So according to Equation (9), the capacitance change can be obtained:

(17) ΔC=πt[Re(ε˜mix)−εo]2m2∑i=1m[(2i−1)t+2mR]qi

The capacitance is only related to the permittivity of media when the structural parameters of the double coil and the excitation alternating current are determinate. Based on the difference in relative permittivity between hydraulic oil, air and water, the capacitance detection mode can distinguish between droplets and bubbles in the oil by using the fringe effect of the capacitor.

## 4. Experiments and Discussion

**Figure 5:**
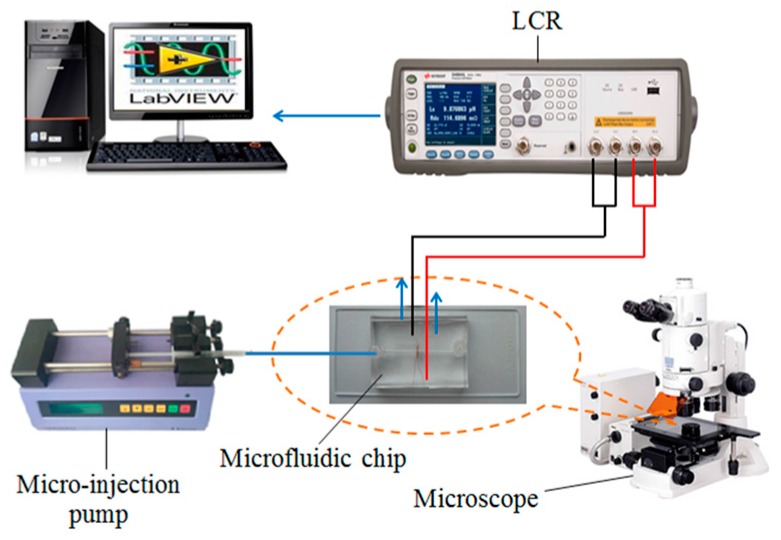
Schematic diagram of impedance detection system.

The impedance detection system is shown in Figure 5. It consists of a micro-injection pump (Harvard Apparatus B-85259, Harvard Apparatus, Holliston, MA, USA), a microscope (Nikon AZ100, Nikon, Tokyo, Japan), a microfluidic chip, an LCR meter (Agilent E4980A, Agilent Technologies Inc., Bayan Lepas, Malaysia) and a computer with LabVIEW software.

### 4.1. Sample Preparations

In our experiment, each type of particle with different sizes was detected. For iron particles, sizes ranging from 100 to 110 μm, 70 to 80 μm and 40 to 50 μm were used. Four milligram iron particles of each size (Hefei Shatai Mechanical and electrical technology Co., Ltd., Hefei, China) were weighed using a precision balance (Precisa XS225A, Precisa Gravimetrics AG, Luzern, Switzerland). They were mixed with 100 mL of marine hydraulic oil (The Great Wall L-HM 46, Sinopec Lubricant Co., Ltd., Beijing, China) and oscillated uniformly using an oscillator (IKA S25, IKA, Staufen, Germany). The same process was also used for 4 mg copper particles ranging from 140 to 150 μm, 120 to 130 μm and 110 to 120 μm. For each particle size, we placed 1 mL particle samples in a plastic test tube for measurement. Both iron particles and copper particles are roughly spherical in shape.

To generate water droplets, 995 μL of hydraulic oil mixed with 5 μL of water was poured into a 1 mL sealed plastic tube. The plastic tube was first oscillated in the oscillator for 0.5 min and then oscillated by an ultrasonic oscillator (DSA200-SK, 200W, 40 KHz, Desen, Fuzhou, China) for 1 min. Via this method, water droplets with a diameter of 210 to 220 μm were generated. When the oscillating time of the ultrasonic oscillator was increased to 1.5 min, the diameters of water droplets were reduced to values from 190 to 200 μm; when the oscillating time was increased to 5 min, the diameters of water droplets were further reduced to values from 100 to 110 μm. Using the same method, 10 μL of air was first injected into 990 of μL hydraulic oil using a pipette, and the plastic tube was sealed quickly. The mixture was oscillated in the oscillator for 1 min and then oscillated by an ultrasonic oscillator for 1 min; then, bubbles with diameters from 280 to 300 μm were generated. When the oscillating time of the ultrasonic oscillator was increased to 1.5 min, bubbles with diameters from 260 to 280 μm were generated; when the oscillating time was increased to 3 min, the diameters of bubbles were reduced to values from 180 to 200 μm.

### 4.2. Inductance Detection Experiments

The droplets and bubbles cannot be detected in inductance detection mode; thus, we only detected iron and copper particles. The double-coil sensor was connected according to Figure 2 and the detection system was built according to Figure 5. To confirm the measurements and demonstrate the utility of the sensor, both iron and copper particles of each size were detected at different excitation frequencies and for different numbers of turns.

The excitation voltage of the LCR meter was set to 2 V; the excitation frequency was set from 1.2 to 2 MHz with a step of 0.2 MHz, and each coil had 20 turns (the quality factors of the 20 turns coil measured by the LCR meter are 6.74, 7.63, 8.47, 8.86, 9.24, 9.59 and 9.94 at the frequencies of 1.2, 1.4, 1.6, 1.7, 1.8, 1.9 and 2.0 MHz, respectively). The flux of the micro-injection pump was set to 40 μL/min. Then, iron particle samples with sizes ranging from 100 to 110 μm and copper particle samples with sizes ranging from 140 to 150 μm were instilled into the detection chip, respectively, the micro-injection pump was turned on and the experiments were started.

**Figure 6:**
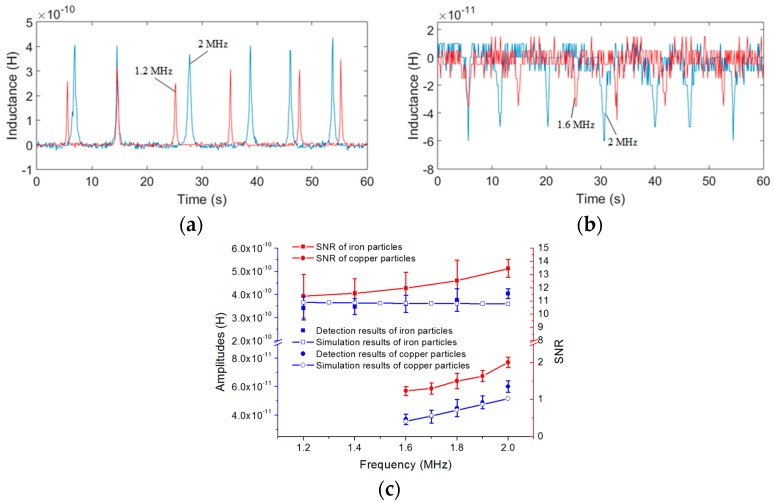
Inductance detection results and theoretical inductance changes at different excitation frequencies (each single-layer coil has 20 turns): (**a**) detection results of iron particles with sizes ranging from 100 to 110 μm, excitation frequency of the red line was 1.2 MHz; excitation frequency of the blue line was 2.0 MHz; (**b**) detection results of copper particles with sizes ranging from 140 to 150 μm, excitation frequency of the red line was 1.6 MHz; excitation frequency of the blue line was 2.0 MHz; (**c**) comparison between the experimental results and the analytical predictions at different excitation frequencies, the relative permeability of iron and copper particle were 500 and 1, respectively; and the conductivity of iron and copper particle were 9.93 × 10^6^ S/m and 5.71 × 10^7^ S/m, respectively.

The detection results of iron and copper particles are shown in Figure 6a,b, respectively, and the comparison between the experimental results and the analytical predictions at different excitation frequencies are shown in Figure 6c. The analytical predictions were conducted using MATLAB to verify the influence of the excitation frequency and the number of turns on the inductance change based on Equation (8), the mutual inductance *M* was calculated from Equation (1) by measuring the *L* and *L*_eq_ of the coil with different turns, as shown in Table 1.

It is easy to observe that the signals produced by the 2 MHz excitation (blue line) are bigger than the signals produced by the 1.2 MHz/1.6 MHz excitation (red line). In Figure 6c, we took the average of 10 detected signal amplitudes as the result of each excitation frequency. The detected signal amplitudes increase with the excitation frequency and the noise is constant in different frequencies; thus, the signal-to-noise ratio (SNR) also increases with the excitation frequency. According to the detection results, we know that an excitation frequency of 2 MHz is better than any other excitation frequency.

**Figure 7:**
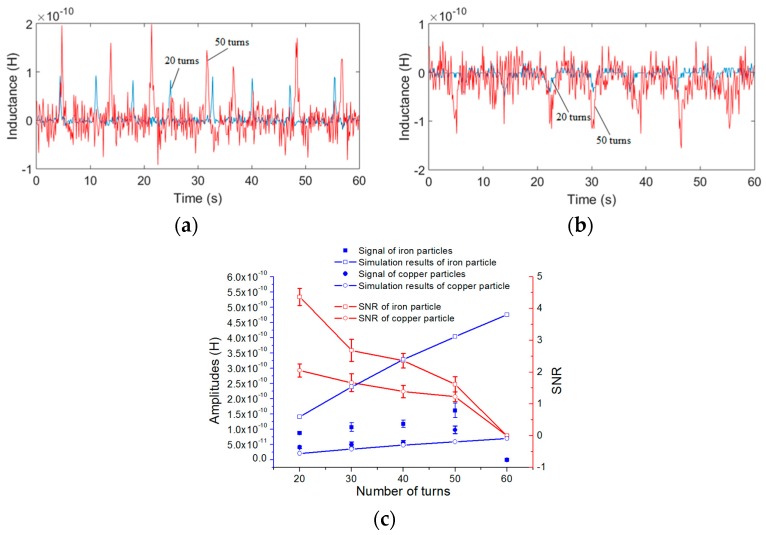
Inductance detection results and theoretical inductance changes for different numbers of turns (excitation frequency was 2 MHz): (**a**) detection results of iron particles with sizes ranging from 70 to 80 μm, red line was 50 turns and blue line was 20 turns; (**b**) detection results of copper particles with sizes ranging from 120 to 130 μm, red line was 50 turns and blue line was 20 turns; (**c**) comparison between the experimental results and the analytical predictions for different numbers of turns.

Therefore, excited by a 2 MHz alternating current, iron particles with sizes ranging from 70 to 80 μm and copper particles with sizes ranging from 120 to 130 μm were detected for different numbers of turns of each single-layer coil. The inductance detection results and analytical predictions are shown in Figure 7.

Figure 7a,b show that the inductance amplitudes of coils with 50 turns (red line) are bigger than the amplitudes of coils with 20 turns (blue line); the same is true for the noise. In Figure 7c, we also took the average of 10 detected signal amplitudes as the result for each number of turns; it is shown that both the amplitudes and the noise increase with the number of turns. However, for a coil with 60 turns, the noise is much bigger than the signal amplitude, the signal is submerged by the noise, and thus no signal can be detected in the coil with 60 turns. Because the noise increases at a faster rate than the signal, the SNR decreases with the number of turns. Thus, from the results, we know that the single-layer coil with 20 turns is better than the other coils.

**Figure 8:**
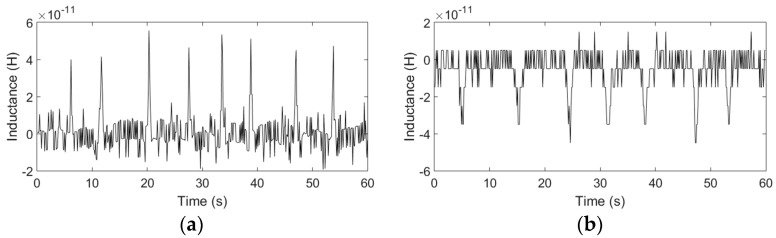
Detection results of iron and copper particles, the frequency was 2 MHz and each coil had 20 turns: (**a**) iron particles with sizes ranging from 40 to 50 μm; (**b**) copper particles with sizes ranging from 110 to 120 μm.

According to the above experimental results, the optimal excitation frequency and the optimal number of turns has been obtained. Therefore, with 2 MHz excitation, by using the coil with 20 turns, iron particles with sizes ranging from 40 to 50 μm and copper particles with sizes ranging from 110 to 120 μm can be successfully detected, as shown in Figure 8.

For both iron and copper particles, the theoretical inductance changes predicted by using (8) are in good agreement with the experimental results: the inductance change of the double coil is related to the number of turns of the single coil *N*, the excitation frequency and the radius of particles *r*. Firstly, the analytical predictions show that the excitation frequency has little effect on the detection of iron particles, but for the copper particles, the inductance amplitude increases with the frequency; the trend is consistent with the results of the theoretical results. In further experiments, when the excitation frequency is lower than 1.6 MHz, the detection signal of copper particles with sizes ranging from 140 to 150 μm cannot be identified, but the iron particles in the size of 100 to 110 μm can be easily detected from 0.05 to 2 MHz. Secondly, both the amplitudes and the noise increase with the number of turns, and the theoretical results show the same trend of inductance amplitudes, but because the noise increases at a faster rate than the signal, the SNR decreases with the number of turns. However, since the analytical predictions are carried out in the ideal case, the inductance amplitude obtained by (8) is slightly different from the experimental results, especially for different turns, because the noise increases sharply with the number of turns, the experimental error also increases. Moreover, the metal particles used in the experiments are not standard spherical, which also increases the experimental error. Lastly, the inductance amplitudes increase with the particle sizes, as shown in Table 2 (a conclusion of the inductance experiments with different particle sizes), but the rate of increase of the amplitudes is not linear compared to that of the particle radius. Since the size of particles fluctuates within a certain range, there are some fluctuations of the signal amplitudes in the signal figures.

### 4.3. Capacitance Detection Experiments

In the capacitance detection mode, the double-coil sensor was connected according to Figure 3 and the detection system was built according to Figure 5; then, the water droplets and bubbles were detected. As in inductance detection, both water droplets and bubbles of each size were detected at different excitation frequencies and for different numbers of turns.

The excitation voltage of the LCR meter was set to 2 V; the excitation frequency was set from 0.3 to 1.9 MHz with a step of 0.2 MHz. The flux of the micro-injection pump was set to 40 μL/min. Then droplet samples with sizes ranging from 210 to 220 μm and bubble samples with sizes ranging from 280 to 300 μm were instilled into the detection chip, respectively, the micro-injection pump was turned on and the experiments were started.

**Figure 9:**
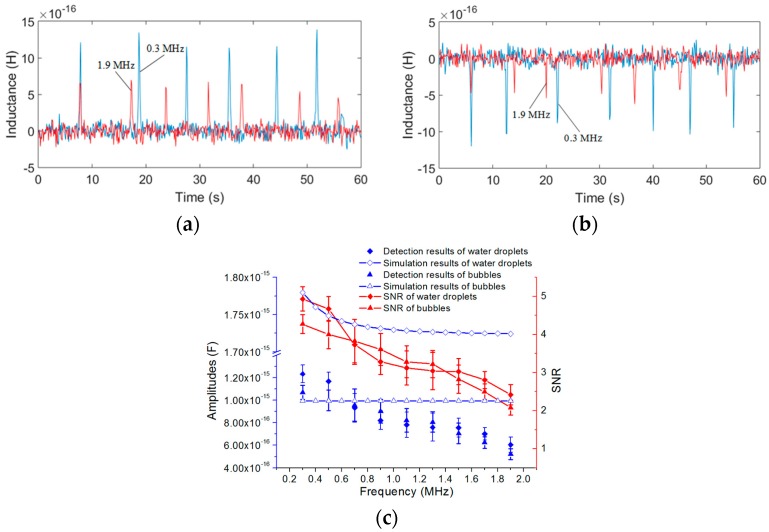
Capacitance detection results and theoretical capacitance changes at different excitation frequencies (each coil has 20 turns): (**a**) detection results of water droplets with sizes ranging from 210 to 220 μm, excitation frequency of the red line was 1.9 MHz; excitation frequency of the blue line was 0.3 MHz; (**b**) detection results of bubbles with sizes ranging from 280 to 300 μm, excitation frequency of the red line was 1.9 MHz; excitation frequency of the blue line was 0.3 MHz; (**c**) comparison between the experimental results and the analytical predictions at different excitation frequencies, the conductivity of water droplet, bubble and oil were 1 × 10^−4^ S/m, 0 S/m and 8 × 10^−10^ S/m, respectively.

The detection results of water droplets and bubbles are shown in Figure 9a,b, respectively, and the comparison between the experimental results and the analytical predictions at different excitation frequencies are shown in Figure 9c. The same as inductance detection, the analytical predictions were conducted using MATLAB to verify the influence of the excitation frequency and the number of turns on the capacitance change based on Equation (17).

The results in Figure 9a,b show that the signals produced by the 0.3 MHz excitation (blue line) are bigger than the signals produced by the 1.9 MHz excitation (red line). In Figure 9c, we took the average of 10 detected signal amplitudes as the result at each excitation frequency. The detected signal amplitudes decrease with the excitation frequency and the noises are constant at different frequencies; thus, the SNR also decreases with the excitation frequency. According to the detection results, we know that an excitation frequency of 0.3 MHz is better than any other excitation frequency.

**Figure 10:**
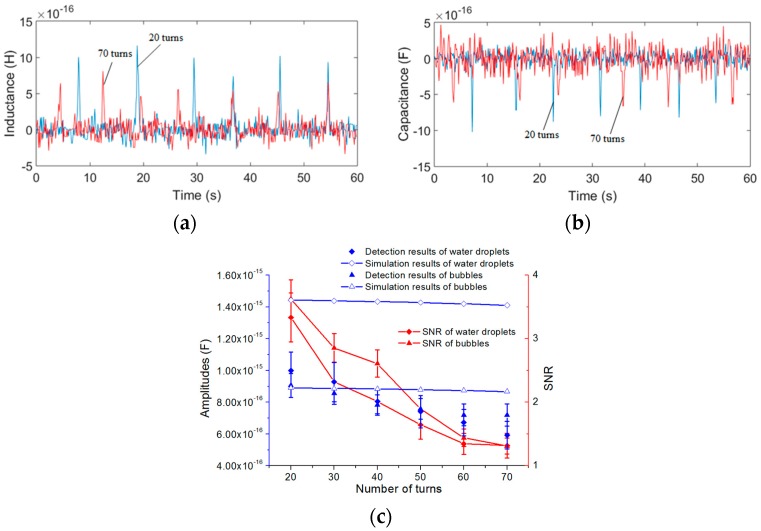
Capacitance detection results and theoretical inductance changes for different numbers of turns (frequency was 0.3 MHz): (**a**) detection results of water droplets with sizes ranging from 190 to 200 μm, red line was 70 turns and blue line was 20 turns; (**b**) detection results of bubbles with sizes ranging from 260 to 280 μm, red line was 70 turns and blue line was 20 turns; (**c**) comparison between the experimental results and the analytical predictions for different numbers of turns.

Therefore, excited by a 0.3 MHz alternating current, water droplets with sizes ranging from 190 to 200 μm and bubbles with sizes ranging from 260 to 280 μm were detected for different numbers of turns of each single-layer coil. The inductance detection results and analytical predictions are shown in Figure 10.

Figure 10a,b show that the capacitance amplitudes of the coil with 20 turns (blue line) are bigger than the amplitudes of the coil with 70 turns (red line). In Figure 10c, we also took the average of 10 detected signal amplitudes as the result for each number of turns; it is shown that the amplitudes decrease with the number of turns, while the noise increases with the number of turns. Therefore, the SNR decreases with the number of turns. From the results we know that the single-layer coil with 20 turns is better than the other coils.

**Figure 11:**
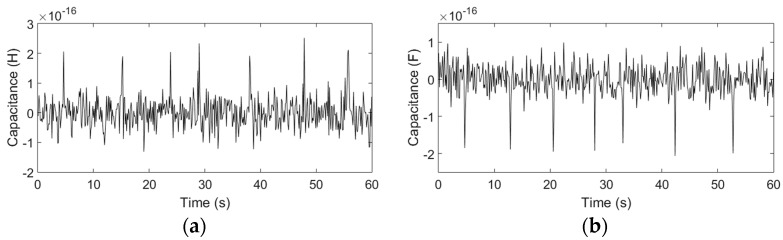
Detection results of water droplets and bubbles, the frequency was 0.3 MHz and each coil had 20 turns: (**a**) water droplets with sizes ranging from 100 to 110 μm; (**b**) bubbles with sizes ranging from 180 to 200 μm.

According to the above experimental results, the optimal excitation frequency and the optimal number of turns have been obtained. Therefore, with 0.3 MHz excitation, by using the coil with 20 turns, water droplets with sizes ranging from 100 to 110 μm and bubbles with sizes ranging from 180 to 200 μm can be successfully detected, as shown in Figure 11.

For both water droplets and bubbles, the trend of the experimental results is consistent with the theoretical analysis: the capacitance change of the double-coil is mainly related to the relative permittivity, the detection volume, the frequency and the radius of particles *r*. The analytical predictions based on (17) show that both the excitation frequency and the number of turns have little effect on the detection of bubbles, but for water droplets, the capacitance amplitude decreases slightly with the frequency and the number of turns. However, in the experiments, the detected signal amplitudes decrease slightly with both the excitation frequency and the number of turns, and the capacitance change predictions are a little larger than the experimental results. The main reason is that the theoretical analysis is based on an approximate ideal model, but in this paper, the capacitor consisted of two equivalent parallel plates (two single-layer coils) is very special, and the construction of a precise theoretical model for the special capacitor is difficult and needs a lot of length to discuss, after reviewing a large number of literature, the results calculated by the theoretical model used in this paper are the closest to the experimental results. In addition, micro capacitance detection is susceptible to external ambient noise, and the measurement noise increases with the number of turns, these factors also cause the experimental errors. The difference in the relative permittivity between air ($\varepsilon_{a}$ = 1) and hydraulic oil ($\varepsilon_{o}$ = 2.6) is smaller than that between water ($\varepsilon_{w}$ = 80) and hydraulic oil ($\varepsilon_{o}$ = 2.6); thus, the capacitor is more sensitive to water droplets than to bubbles. When the excitation frequency is lower than 0.15 MHz, the detection signal of both bubbles and water droplets cannot be identified. The capacitance amplitudes also increase with the particle diameter, as shown in Table 3 (a conclusion of the capacitance experiments with different particle sizes), but the rate of increase of the amplitudes is not linear compared to that of the particle radius. Since the size of particles fluctuates within a certain range, there are some fluctuations of the signal amplitudes in the signal figures.

## 5. Conclusions

A novel impedance sensor based on a microfluidic chip has been presented for the detection of particle contamination in hydraulic oil. The sensor can detect not only ferromagnetic and non-ferromagnetic particles in oil as an inductance sensor but also water droplets and air bubbles in oil as a capacitance sensor. The design, fabrication, and the simulation of analytical modeling are discussed in detail. In our experiments, the excitation frequency and the number of coil turns of the sensor were studied. For the inductance detection, the excitation frequency was varied in the range of 1.2 to 2 MHz for iron particle detection and in the range of 1.6 to 2 MHz for copper particle detection. The inductance signals were found to increase with the excitation frequency and the noise is constant at different frequencies; both the inductance signals and the noise increase with the number of coil turns, but because the noise increases at a faster rate than the signal, the SNR decreases with the number of coil turns. We have detected 40 μm iron particles and 110 μm copper particles successfully using the two coils with 20 turns at the excitation frequency of 2 MHz. For the capacitance detection, capacitance signals decrease with the excitation frequency and the noise is constant at different frequencies; the capacitance signals decrease with the number of coil turns, and the noise increases; thus, the SNR decreases with the number of coil turns. We have detected 100 μm water droplets and 180 μm bubbles successfully using the two coils with 20 turns at the excitation frequency of 0.3 MHz. The inductance detection results are in good agreement with the analytical prediction, but there is a small error between capacitance detection results and the analytical prediction, and the detailed error analysis is discussed, so a more precise theoretical model should be built in the future. When the inductance detection mode is in operation, the water droplets and bubbles in the oil do not affect the detection results, and due to the difference in excitation frequency, when the capacitance detection mode is working, the iron particles and copper particles in the oil can hardly produce an influence on the test results, so the two detection modes can run independently and effectively. However, the detection accuracy of these two detection modes is limited currently, and the flux is not high, so our next major task is to optimize the chip and detection system to improve the detection accuracy and throughput. The impedance sensor presented in this paper can detect four types of particle contamination in hydraulic oil, which has important implications for the working condition monitoring and fault diagnosis of hydraulic systems.

## Figures and Tables

**Table 1:** This is a table. Tables should be placed in the main text near to the first time they are cited.

Parameters	*N* = 20	*N* = 30	*N* = 40	*N* = 50	*N* = 60
*L* (μH)	1.4030	2.8435	5.3643	9.5557	15.8967
*L*_eq_ (μH)	1.1037	2.5144	5.0266	9.0852	15.4097
*M* (μH)	0.8044	2.1835	4.6889	8.6147	14.9227
*M*/*L*	0.5733	0.7679	0.8741	0.9015	0.9387

**Table 2:** The relationship between particle sizes and inductance amplitudes, the excitation frequency is 2 MHz and the number of coil turns is 20 turns.

Iron Particle Sizes (μm)	Average Amplitude (H)	Copper particle Sizes (μm)	Average Amplitude (H)
40 to 50	4.4 × 10^−11^	110 to 120	3.8 × 10^−11^
70 to 80	8.7 × 10^−11^	120 to 130	4. 1× 10^−11^
100 to 110	4.04 × 10^−10^	140 to 150	5.9 × 10^−11^

**Table 3:** The relationship between particle sizes and capacitance amplitudes, the excitation frequency is 0.3 MHz and the number of coil turns is 20 turns.

Water Droplet Sizes (μm)	Average Amplitude (H)	Bubble Sizes (μm)	Average Amplitude (H)
100 to 110	2.1 × 10^−16^	180 to 200	1.9 × 10^−16^
190 to 200	1.02 × 10^−15^	260 to 280	7.7 × 10^−16^
210 to 120	1.23 × 10^−15^	280 to 300	9.6 × 10^−16^

## References

[1] Zhang H., Huang W., Zhang Y., Shen Y., Li D. (2012). Design of the microfluidic chip of oil detection. Appl. Mech. Mater..

[2] Tucker J.E., Schultz A., Lu C., Sebok T., Holloway C., Tankersley L.L., Reintjes J. Lasernet fines optical wear debris monitor. Proceedings of the International Conference on Condition Monitoring.

[3] Edmonds J., Resner M.S., Shkarlet K. Detection of precursor wear debris in lubrication systems. Proceedings of the 2000 IEEE Aerospace Conference Proceedings.

[4] Toms A., Toms A. (2010). Oil analysis and condition monitoring. Chemistry and Technology of Lubricants.

[5] Abouel-Kasem A., Alturki F.A., Ahmed S.M. (2011). Fractal analysis of cavitation eroded surface in dilute emulsions. J. Tribol..

[6] Zou J., Wang C., Fu X. (2012). Design and experiment of on-line bubble elimination valve for hydraulic oil. Hydraul. Pneum. Seals.

[7] Sun W. (2011). Quantitative estimation technique for wear amounts by real time measurement of wear debris in lubricating oil. Adv. Mater. Res..

[8] Wu T., Wu H., Du Y., Peng Z. (2013). Progress and trend of sensor technology for on-line oil monitoring. Sci. China Technol. Sci..

[9] Khandaker I.I., Glavas E., Jones G.R. (1993). A fibre-optic oil condition monitor based on chromatic modulation. Meas. Sci. Technol..

[10] Kwon O.K., Kong H.S., Han H.G., Yoon E.S., Myshkin N.K., Markova L.V., Semeniouk M.S. (2000). On-line Measurement of Contaminant Level in Lubricating Oil. U.S. Patent.

[11] Zhang J., Drinkwater B.W., Dwyer-Joyce R.S. (2006). Monitoring of lubricant film failure in a ball bearing using ultrasound. J. Tribol..

[12] Du L., Zhe J., Carletta J., Veillette R., Choy F. (2010). Real-time monitoring of wear debris in lubrication oil using a microfluidic inductive Coulter counting device. Microfluid. Nanofluid..

[13] Du L., Zhe J. (2011). A high throughput inductive pulse sensor for online oil debris monitoring. Tribol. Int..

[14] Zhang H., Zhang Y., Zhang C., Shen Y., Pan X., Li D. (2012). Detection of micro debris based on spatial micro solenoid inductance. Mach. Tool Hydraul..

[15] Zhang X., Zhang H., Sun Y., Chen H., Zhang Y. (2014). Research on the output characteristics of microfluidic inductive sensor. J. Nanomater..

[16] Du L., Zhu X., Han Y., Zhan L., Zhe J. (2013). Improving sensitivity of an inductive pulse sensor for detection of metallic wear debris in lubricants using parallel LC resonance method. Meas. Sci. Technol..

[17] Murali S., Xia X., Jagtiani A.V., Carletta J., Zhe J. (2009). Capacitive coulter counting: Detection of metal wear particles in lubricant using a microfluidic device. Smart Mater. Struct..

[18] Zheng Y., Shojaei-Baghini E., Wang C., Sun Y. (2013). Microfluidic characterization of specific membrane capacitance and cytoplasm conductivity of singlecells. Biosens. Bioelectron..

[19] Li M., Zhao K., Song Y., Pan X. (2013). Microfluidic capacitance sensor for detecting metal wear debris in lubrication oil. Dalian Haishi Daxue Xuebao.

[20] Zhu X., Du L., Zhe J. (2014). An integrated lubricant oil conditioning sensor using signal multiplexing. J. Micromech. Microeng..

[21] Wu Y., Zhang H., Zeng L., Chen H., Sun Y. (2016). Determination of metal particles in oil using a microfluidic chip-based inductive sensor. Instrum. Sci. Technol..

[22] Liu B. (2013). Measuring core and ferrite saturable inductance. Foreign Electron. Meas. Technol..

[23] Zhou D., Wang J., Zhang Q., Wu J., Zhang H. (2013). Research on sensing mechanism of ferromagnetic component flaw using pulsed eddy current testing. Yiqi Yibiao Xuebao.

[24] Zhang X. (2014). Study on Metal Particle Magnetization in Harmonic Field. Ph.D. Thesis.

[25] Fan H., Zhang Y., Cheng Y., Ren G. (2010). Effect of the radial distribution of the wear debris position on the testing results of inductive wear debris sensor. Chin. J. Sens. Actuators.

[26] Liu E. (2017). Research on the Detection of Various Pollutants in Marine Hydraulic Oil in an Integrated Chip. Master Thesis.

[27] Palmer H.B. (1937). The capacitance of a parallel-plate capacitor by the Schwartz-Christoffel transformation. Electr. Eng..

[28] Hosseini M., Zhu G., Peter Y.A. (2007). A new formulation of fringing capacitance and its application to the control of parallel-plate electrostatic micro actuators. Analog Integr. Circuits Signal Process..

[29] Kambali P.N., Pandey A.K. (2016). Capacitance and force computation due to direct and fringing effects in MEMS/NEMS arrays. IEEE Sens. J..

[30] Nishiyama H., Nakamura M. (1993). Capacitance of disk capacitors. IEEE Trans. Compon. Hybrids Manuf. Technol..

[31] Morgan H., Sun T., Holmes D., Gawad S., Green N.G. (2006). Single cell dielectric spectroscopy. J. Phys. D Appl. Phys..

